# A topology-based method to mitigate the dosimetric uncertainty caused by the positional variation of the boost volume in breast conservative radiotherapy

**DOI:** 10.1186/s13014-017-0801-4

**Published:** 2017-03-20

**Authors:** Peng-Yi Lee, Chih-Yuan Lin, Shang-Wen Chen, Chun-Ru Chien, Chun-Nan Chu, Hsiu-Ting Hsu, Ji-An Liang, Ying-Jun Lin, An-Cheng Shiau

**Affiliations:** 1 Department of Radiation Oncology, China Medical University Hospital, 2nd Yu-De Road, North District, Taichung City, Taiwan; 2 Department of Medicine, China Medical University, Taichung, Taiwan; 3 Department of Medicine, Taipei Medical University, Taipei, Taiwan; 4 Department of Biomedical Imaging and Radiological Sciences, National Yang-Ming University, Taipei, Taiwan

**Keywords:** Breast cancer, Adjuvant radiotherapy, Tumor bed uncertainty, Simultaneous integrated boost

## Abstract

**Background:**

To improve local control rate in patients with breast cancer receiving adjuvant radiotherapy after breast conservative surgery, additional boost dose to the tumor bed could be delivered simultaneously via the simultaneous integrated boost (SIB) modulated technique. However, the position of tumor bed kept changing during the treatment course as the treatment position was aligned to bony anatomy. This study aimed to analyze the positional uncertainties between bony anatomy and tumor bed, and a topology-based approach was derived to stratify patients with high variation in tumor bed localization.

**Methods:**

Sixty patients with early-stage breast cancer or ductal carcinoma in situ were enrolled. All received adjuvant whole breast radiotherapy with or without local boost via SIB technique. The delineation of tumor bed was defined by incorporating the anatomy of seroma, adjacent surgical clips, and any architectural distortion on computed tomography simulation. A total of 1740 on-board images were retrospectively analyzed. Positional uncertainty of tumor bed was assessed by four components: namely systematic error (SE), and random error (RE), through anterior-posterior (AP), cranial-caudal (CC), left-right (LR) directions and couch rotation (CR). Age, tumor location, and body-mass factors including volume of breast, volume of tumor bed, breast thickness, and body mass index (BMI) were analyzed for their predictive role. The appropriate margin to accommodate the positional uncertainty of the boost volume was assessed, and the new plans with this margin for the tumor bed was designed as the high risk planning target volume (PTV-H) were created retrospectively to evaluate the impact on organs at risk.

**Results:**

In univariate analysis, a larger breast thickness, larger breast volume, higher BMI, and different tumor locations correlated with a greater positional uncertainty of tumor bed. However, BMI was the only factor associated with displacements of surgical clips in the multivariate analysis and patients with higher BMI were stratified as high variation group. When image guidance was aligned to bony structures, the SE and RE of clip displacement were consistently larger in the high variation group. The corresponding PTV-H margins for the high- and low-variation groups were 7, 10, 10 mm and 4, 9, 6 mm in AP, CC, LR directions, respectively. The heart dose between the two plans was not significantly different, whereas the dosimetric parameters for the ipsilateral lung were generally higher in the new plans.

**Conclusions:**

In patients with breast cancer receiving adjuvant radiotherapy, a higher BMI is associated with a greater positional uncertainty of the boost tumor volume. More generous margin should be considered and it can be safely applied through proper design of beam arrangement with advanced treatment techniques.

## Background

Breast conservative therapy including lumpectomy and adjuvant whole breast radiotherapy has become standard treatment for patient with early stage breast cancer [1, 2]. Additional boost dose to the tumor bed via electron or photon beam further improve local control rate [3]. Treatment techniques with beam arrangement using the tangent angles can have a limited dose to the ipsilateral lung and the contralateral breast but are difficult to generate a concave dose distribution conforming to the breast target. Advanced techniques like intensity-modulated radiation therapy (IMRT), tomotherapy, and volumetric intensity-modulated arc radiation therapy (VMAT) offer the ability to provide a more sophisticated process through the inverse planning procedure, generating a more conformal dose distribution to the breast target, sparing the high dose region to the anterior heart, and improving dose homogeneity [4–8]. Through proper design of beam arrangement with advanced treatment techniques, plans possessing a concave dose distribution with limited doses to the critical heart, lung, and contralateral breast should be achievable. In our institution, most of patients received boost dose with a simultaneous integrated boost (SIB) modulated technique. With the use of SIB technique, accuracy of dose delivery to the tumor bed is essential for proper treatment. Surgical clips in the peripheral of the tumor bed have been evaluated in several studies as the fiducial marker to improve the boost accuracy [9–12]. A study from Italy investigated the role of surgical clips in defining the clinical target volume (CTV) for partial breast irradiation. They conclude that surgical clips are essential and six or more increase the accuracy of tumor bed localization [11]. However, the position of tumor bed kept changing during the treatment course as the treatment position was aligned to bony anatomy and there is no consistency in positioning between bony structures and surgical clips during daily image guidance [12].

The aim of the current study was to compare the positional uncertainty between bony anatomy and surgical clip of tumor bed. In addition, the impact of body-mass factors (BMF) on magnitude of surgical clip displacements was determined. A topology-based approach was derived to stratify patients with high variation of tumor-bed position, and the corresponding margin of high risk planning target volume (PTV-H) was evaluated to accommodate the dose coverage of tumor-bed. This method would provide an accurate, efficient, and cost-effective clinical application.

## Methods

### Patients and treatment concept

With the approval of the local institutional review board, 60 consecutive patients with early-stage, node-negative breast cancers diagnosed in between 2014 and 2015 were enrolled. Forty-eight patients were victims of invasive cancer, after partial mastectomy and sentinel lymph node biopsy, all received adjuvant whole breast radiotherapy using a SIB technique with median dose of 5880 cGy to tumor bed and 5040 cGy to whole breast in 28 fractions under daily image guidance. The other 12 patients with carcinoma in situ underwent adjuvant whole breast radiotherapy of 5040 cGy in 28 fractions without local boost. Surgical clips marking the position of the tumor bed had been implanted at the time of breast-conserving surgery. Patient-related factors consisted of age, tumor location, and body mass factors were acquired from chart record and simulation computed tomographic (CT) images. Body mass factors included body weight, body height, body mass index (BMI), volume of breast, volume of tumor bed, and breast thickness. Breast thickness was defined by the distance between the chest wall and the skin surface at the level of the nipple [13]. Detail of patient characters was illustrated in Table 1.

**Table 1:** Patient and treatment characteristics (*N* = 60)

Characteristics		Number (%)
Age (years)	Median (Range)	50 (33-65)
Laterality	Right/Left	26 (43.3%)/34 (56.7%)
Histology	Ductal carcinoma in situ	12 (20%)
	Invasive carcinoma	48 (80%)
Axillary nodal status	Negative	60 (100%)
Tumor bed volume (ml)	Median (Range)	24.6 (4.4 – 103.9)
Whole breast volume (ml)	Median (Range)	429.2 (174.1 – 1205.3)
Breast thickness (cm)	Median (Range)	3.01 (1.3 – 6.4)
Body Height (BH) (cm)	Median (Range)	158 (147 – 166.8)
Body Weight (BW) (kgs)	Median (Range)	57.1 (43.8 – 89)
Body mass index (BMI)	Median (Range)	23.1 (18.5 – 34.3)
Tumor Location	UIQ	17 (28.3%)
	LIQ	10 (16.7%)
	UOQ	14 (23.3%)
	LOQ	13 (21.7%)
	Sub	6 (10%)

Abbreviations: *UIQ* upper inner quadrant, *LIQ* lower inner quadrant, *UOQ* upper outer quadrant, *LOQ* lower outer quadrant, *Sub* subareola

### Treatment planning

**Fig. 1:**
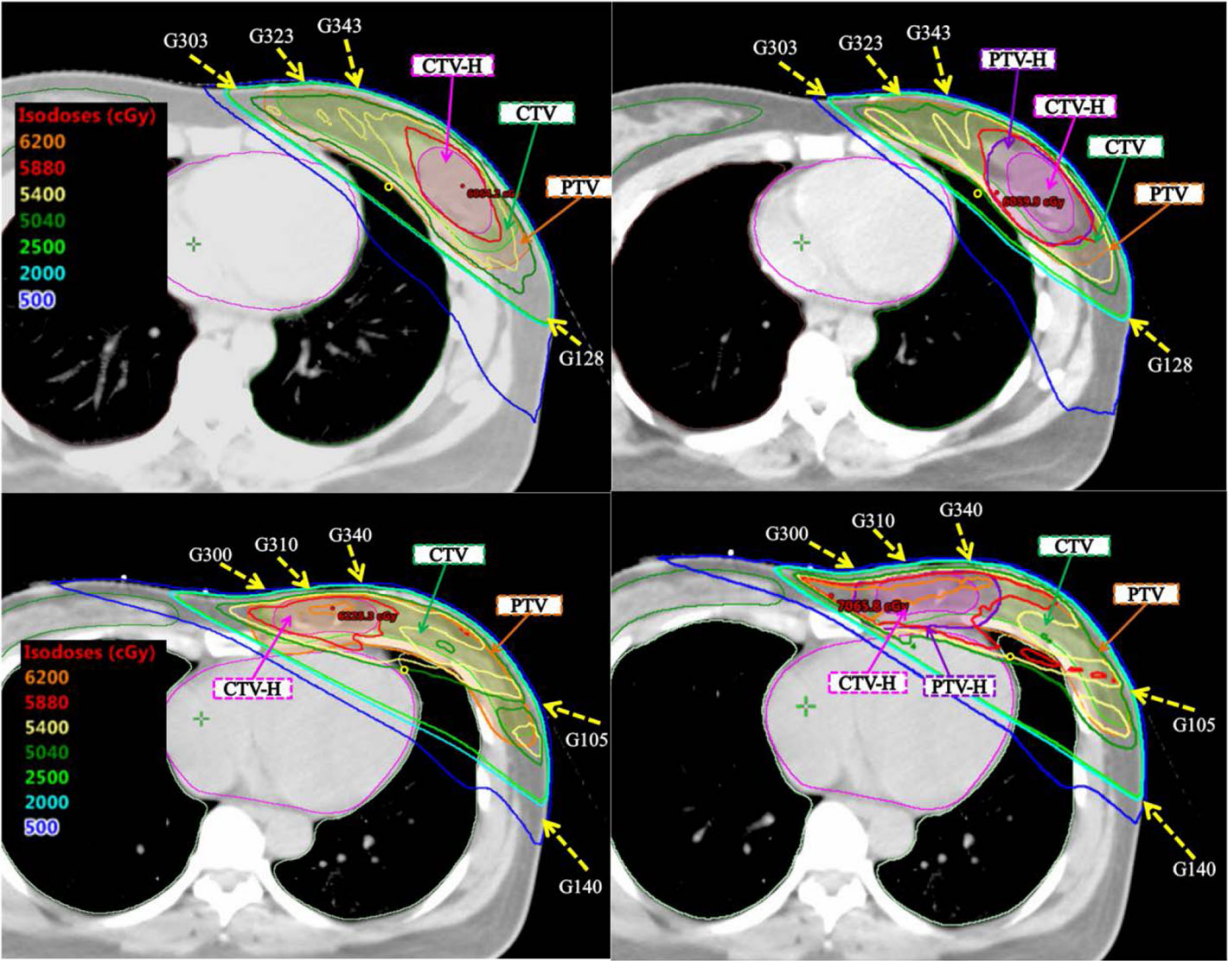
Dose distributions on axial images of CTV-H slices for different patients (up and down), and for the original plan (left) and new plan (right). According to the location of CTV-H, proper beam arrangement was designed for target coverage and critical organ sparing. Through proper design of beam arrangement with advanced treatment techniques, the new plan with boost target (PTV-H) shown limited dose increments to heart and lung

To enhance the accuracy of the daily irradiated position, custom-made immobilization device with ipsilateral arm raised was used for all patients. Following fabrication of the immobilization device, simulation using a CT simulator (HiSpeed NX/i, GE Healthcare, Milwaukee, Wisconsin, USA) was performed with free breathing and 4-mm-slice thickness. Treatment target contouring was completed according to the RTOG consensus atlas [14]. The CTV included the whole lesion-sided breast, and a margin of 5 mm extended isotropically from the CTV to form the PTV. The delineation of tumor bed (CTV-H) was defined by incorporating the anatomy of seroma, adjacent surgical clips and any architectural distortion on the CT image. For those without tumor bed boost, the surgical clips and CTV-Hs were still contoured for localization. All cases with invasive cancer were planned with a concomitant boost delivered in 28 fractions by intensity modulated radiotherapy (IMRT) technique using 6 MV photon beams. The prescribed dose of 5880 cGy to the CTV-H and 5040 cGy to the PTV via 4 to 5-field IMRT was planned (Fig. 1). Two tangential beams were assigned to the PTV and another oblique beams assigned to the CTV-H. The beam angle was adjusted according to the location of tumor bed and critical organs. In the original plan, there was no additional margin added to the CTV-H. All plans were carried out using the Eclipse treatment planning system (Version 11, Varian Medical Systems Inc, Palo Alto, California, USA) with Analytical Anisotropic Algorithm (AAA) for dose calculation.

### Verification of treatment position and data acquisition

All patients were treated under daily image guidance with the Varian Clinac iX Linac (Varian Medical Systems, Palo Alto, California, USA) equipped with an on-line On-Board Imaging (OBI) system. In daily treatment, after patients were set up according to the skin markers, two orthogonal plane images were taken for image registration. Bony structure such as upper sternum and ribs were used as matching positions by two radiation therapists. Then, an attending physician would check and confirm the registered images. We retrospectively registered a total of 1740 daily on-board images to the pretreatment digital reconstruction radiography, and the images were re-matched using surgical clips as surrogates.

The positional uncertainty was compared between bony anatomy and surgical clip of tumor bed, and treatment target displacements for each patient were assessed by four metrics: namely systemic error (SE, ∑), and random error (RE, σ), through three translational directions anterior-posterior (AP), cranial-caudal (CC) and left – right (LR) as well as couch rotation (CR). The ∑ is defined as the variation of the mean error between patients, while the σ represents the root mean square (RMS) of the standard deviations (SD’s) of all patients [15].

### Margin assessment and treatment plan comparison

As a consequence of daily target uncertainty, we disclosed the additional margin, namely PTV-H, of 2.5 ∑ + 0.7 σ should be added around tumor bed by the proposal from Van Herk et al. [16]. We adapted a new plan with the PTV-H for the 48 patients with invasive cancer, and the dose to the target, the heart and the ipsilateral lung were compared between the original and new plans. The beam arrangement and field size were redesigned for the new plan to deliver an adequate dose to the targets and to have a sparing to the critical organs including heart, lung, and contra-lateral breast. For the original plan, the treatment targets were the CTV-H and PTV. For the new plan, the targets were represented by PTV-H and PTV.

### Statistical analysis

The predictive role of associating factors for higher target uncertainty was analyzed, and the median values of the BMFs were used as cut-off points to divide the groups. The univariate analysis was conducted by the Mann-Whitney *U* test or Kruskal-Wallis H test, and the multivariant analysis was carried out by multiple regression analysis via General Linear Model. Comparison between the two treatment plans with and without PTV-H margin were done by the Wilcoxon Signed Rank Test. A two-sided *p* value of < 0.05 was considered statistically significant. All statistical analyses were performed using a commercial software package (SPSS 19.0 for Windows, Chicago, IL, USA).

## Results

Bony alignment is not correlated well with surgical clip of tumor bed in patients with breast cancer receiving dose boost to tumor bed via SIB technique. The displacements of surgical clips and bony anatomy when daily set-up aligning to skin markers were summarized in Table 2. The displacement of surgical clip was numerically larger than bony structures in all orientations except RE in the CC direction. When aligning to bony anatomy with daily image guidance, the SE and RE of surgical clips in AP, CC, LR directions were 2.0, 3.3, 2.7 mm and 1.6, 2.2, 1.9 mm, and the rotational SE and RE was 1.2 and 1.0°, respectively.

**Table 2:** Summary of set-up errors

Error		AP (mm)	CC (mm)	LR (mm)	CR (degree)
Aligning to skin markers					
Bony structure	SE (∑)	1.6	2.5	2.0	0.7
	RE (σ)	1.3	2.6	1.6	0.8
Surgical clip	SE (∑)	2.1	2.6	3.2	1.6
	RE (σ)	1.6	2.4	2.1	1.2
Aligning to bony structures					
Surgical clip	SE (∑)	2.0	3.3	2.7	1.2
	RE (σ)	1.6	2.2	1.9	1.0

Abbreviations: *AP* anterior-posterior, *CC* cranial-caudal, *LR* left-right, *CR* couch rotation; *SE* systematic error, *RE* random error

Age, tumor location, and BMFs including body mass index, volume of the breast, volume of the tumor bed, and breast thickness were potentially attributable to the treatment target uncertainty. We analyzed these factors to evaluate their predictive role. In the univariate analysis, larger breast thickness had correlation with greater AP-SE (*p* = 0.038) and AP-RE (*p* = 0.006), larger breast volume was associated with greater AP-RE (*p* = 0.012), higher BMI had significantly larger AP-RE (*p* = 0.001) and CC-RE (*p* = 0.007), and different tumor locations also had influence on AP-SE (*p* = 0.039) and AP-RE (*p* = 0.011). However, the multivariate analysis showed that BMI was the only factor having impact on tumor bed displacement, in AP-RE (*p* = 0.025), CC-RE (*p* = 0.02), and LR-RE (*p* = 0.001). The details are summarized in Table 3.

**Table 3:**
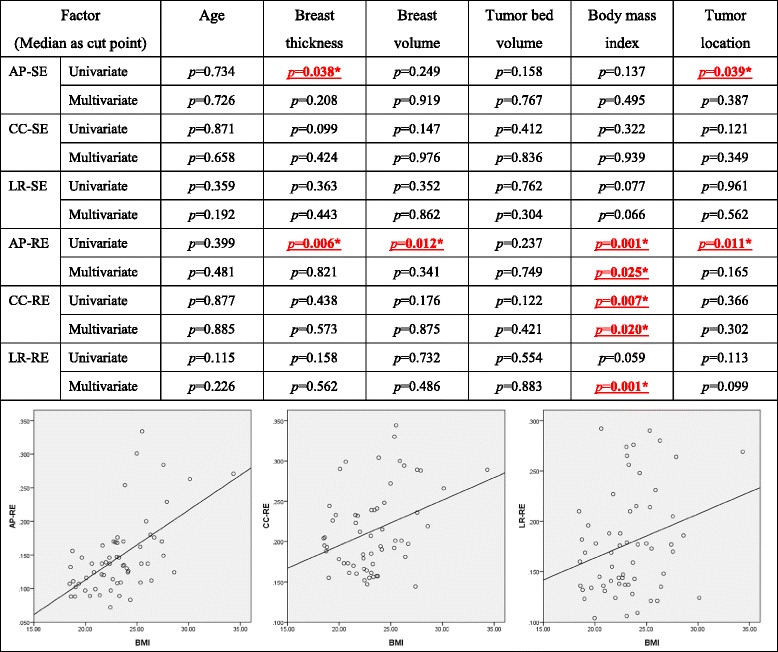
Univariate and multivariate analysis of potential associating factors for larger surgical clip displacement under IGRT

Abbreviations: AP = anterior-posterior; CC = cranial-caudal; LR = left-right; SE = systematic error; RE = random error; * *p <0.05*

According to these results, the 30 patients with higher BMI (≥ median value: 23.1) were defined as high variation group for missing boost volume, and the others with lower BMI were referred as low variation group. When positioning was guided with bony structures by on-line image guidance, the SE’s of surgical clips were 2.4 mm, 3.4 mm, 3.4 mm, 1.3° for high variation group in AP, CC, LR directions and CR, whereas 1.4 mm, 3.2 mm, 1.8 mm, 1.0° for low variation group. The RE were 1.9 mm, 2.4 mm, 2 mm, 1.1° for high variation group, while 1.2 mm, 2 mm, 1.6 mm, 0.9° for low variation group. To guarantee that 90% of patients will receive a minimum cumulative dose to tumor bed of at least 95% of the prescribed dose, the corresponding PTV-H margins around tumor bed according to Van Herk’s formula [16] were 7 mm, 10 mm, and 10 mm in AP, CC, LR directions for high variation group. For low variation group, the corresponding margins could be safely reduced to 4 mm, 9 mm, and 6 mm. The results are listed in Table 4.

**Table 4:** Positioning error and corresponding margin for the boost volume in different groups

	SE (mm)			RE (mm)			Corresponding PTV-H margin (mm)		
	AP	CC	LR	AP	CC	LR	AP	CC	LR
High variation	2.4	3.4	3.4	1.9	2.4	2.0	7.0	10.0	10.0
Low variation	1.4	3.2	1.8	1.2	2.0	1.6	4.0	9.0	6.0

Abbreviations: *AP* anterior-posterior, *CC* cranial-caudal, *LR* left-right, *SE* systematic error; *RE* random error

The dosimetric comparison between the original plan (without PTV-H) and new plan (with PTV-H) are demonstrated in Table 5 and Fig. 1. In the original plan, the treatment target coverage was represented by the volume receiving at least 97% of the prescribed dose, namely the CTV-H and PTV. In the new plan, the targets were represented by PTV-H and PTV. There was no significant difference for mean heart dose or the volume of heart receiving dose larger than 25 Gy regardless of patients in high- or low- variation groups, or tumors in left or right breast. For the ipsilateral lung, mean lung dose, and the volume of lung receiving at least 5 Gy (V5) or 10 Gy (V10) increased significantly for all patient groups. However, the volume of the ipsilateral lung receiving more than 20 Gy (V20) showed significant difference only in patients of high variation group with left side breast cancer.

**Table 5:** Dosimetric comparison between plans with and without PTV-H, data given as median (range) for each parameter

	Original plan (without PTV-H)	New plan (with PTV-H)	*p v*alue
High variation group patient (BMI ≥ 23.1) with left side breast cancer (*N* = 14)			
Tumor bed V_97%_ (%)	99.9 (99, 100)	99.9 (97, 100)	
Whole breast V_97%_ (%)	96.4 (93.2, 98)	97.1 (95.3, 98.2)	
Ipsilateral lung V_5_ (%)	27.4 (19.5, 49.1)	28.5 (20, 48.93)	0.002*
Ipsilateral lung V_10_ (%)	19.4 (12.23, 38.87)	20.1 (12.5, 38.4)	0.006*
Ipsilateral lung V_20_ (%)	15.1 (8.7, 30.6)	15.2 (8.8, 30.5)	0.013*
Ipsilateral lung mean dose (cGy)	847.4 (549, 1537.5)	889.6 (560, 1549.5)	0.001*
Heart V_25_ (%)	2.37 (0.29, 9.13)	2.29 (0.24, 11.1)	0.197
Heart mean dose (cGy)	251.7 (150.7, 543.8)	250.5 (160.7, 649)	0.158
High variation group patient (BMI ≥ 23.1) with right side breast cancer (*N* = 10)			
Tumor bed V_97%_ (%)	99.96 (99.3, 99.99)	99.6 (98.9, 100)	
Whole breast V_97%_ (%)	97.2 (94.7, 98.4)	97.8 (95.5, 98.7)	
Ipsilateral lung V_5_ (%)	30.4 (20.3, 35.6)	31 (20.7, 35.8)	0.005*
Ipsilateral lung V_10_ (%)	20.5 (12.3, 25.9)	20.74 (12.5, 26.1)	0.005*
Ipsilateral lung V_20_ (%)	15.9 (7.6, 20.7)	15.9 (7.5, 21)	0.221
Ipsilateral lung mean dose (cGy)	887 (502, 1080.5)	902 (500, 1089)	0.007*
Heart V_25_ (%)	0 (0, 0)	0 (0, 0)	1
Heart mean dose (cGy)	44.7 (33.8, 66.4)	49.1 (34.6, 66.5)	0.114
Low variation group patient (BMI < 23.1) with left side breast cancer (*N* = 15)			
Tumor bed V_97%_ (%)	100 (98, 100)	100 (97.8, 100)	
Whole breast V_97%_ (%)	97.1 (91.7, 98)	97.2 (92, 98.9)	
Ipsilateral lung V_5_ (%)	29.2 (22.4, 36.2)	29.6 (22.6, 32.3)	0.001*
Ipsilateral lung V_10_ (%)	21.5 (15.6, 27.5)	21.5 (15.9, 27.6)	0.031*
Ipsilateral lung V_20_ (%)	17 (10.9, 21.7)	16.9 (10.9, 21.8)	0.495
Ipsilateral lung mean dose (cGy)	892.3 (668.6, 1148.7)	909.8 (683.6, 1159)	0.003*
Heart V_25_ (%)	1.6 (0.04, 4.66)	1.6 (0.05, 4.86)	0.625
Heart mean dose (cGy)	218.8 (95, 374.2)	218.8 (95.9, 381)	0.073
Low variation group patient (BMI < 23.1) with right side breast cancer (*N* = 9)			
Tumor bed V_97%_ (%)	100 (99.9, 100)	100 (99.8, 100)	
Whole breast V_97%_ (%)	97.4 (94.2, 98.5)	98 (94.7, 98.5)	
Ipsilateral lung V_5_ (%)	30.6 (21.6, 38.4)	31.7 (22.2, 39)	0.008*
Ipsilateral lung V_10_ (%)	21.6 (14.2, 26.3)	22.3 (14.5, 26.6)	0.008*
Ipsilateral lung V_20_ (%)	15.7 (10.4, 19.9)	16.2 (10.7, 19.4)	0.066
Ipsilateral lung mean dose (cGy)	902.3 (623.7, 1099)	929.1 (639.2, 1111)	0.008*
Heart V_25_ (%)	0 (0, 0)	0 (0, 0)	1
Heart mean dose (cGy)	43.1 (22.8, 53)	43.2 (24, 53.2)	0.05

*p <0.05

## Discussion

As far as we know, this is the first study to demonstrate the positional uncertainty of tumor bed in patients with breast cancer when a SIB technique is implicated. In addition, we assessed the corresponding PTV-H margin to stratify patients for the high- and low-variation groups, and to evaluate radiation dose to normal tissue. Our results indicated that the positional uncertainty of tumor bed was prominent and could not be ignored during planning or treatment. Because bony structure is frequently used as a surrogate for daily image-guided radiotherapy, several findings of this study should merit additional attention.

Daily image guidance with two orthogonal kilovoltage (KV) plane images is a time-saving and cost-effective approach, but the correlation of bony anatomy and surgical clip of tumor bed is a major concern. Dozens of studies focusing on clip displacement were in use of accelerated partial breast irradiation technique (APBI). Marco Trovo et al. [9] reported 15 patients undergoing image-guided partial breast irradiation with 3 fiducial markers placed in the tumor bed. Daily orthogonal anterior/posterior and lateral kV-images were taken before each fraction and compared with the digitally reconstructed radiographs (DRRs). When using fiducial markers as the reference, the mean shifts of the treatment target were 0 mm, 1 mm, 0 mm along superior/inferior, right/left, and anterior/posterior directions, meanwhile, the standard deviations were 7 mm, 4 mm, 5 mm, respectively. Park et al. [17] enrolled 26 patients were treated with 3D conformal APBI, each had three or four textured gold intraparenchymal fiducials placed at the periphery of the lumpectomy cavity. The average variation in daily separation between the fiducial pairs from daily megavoltage (MV) images was 3 mm ± 3 mm. Thus, they concluded that fiducial markers are stable throughout the course of APBI, and planning target volume margins when using bony landmarks should be 10 mm and can be reduced to 6 mm if using the fiducials.

Nevertheless, there is no compelling data in the literature in the circumstance of whole breast RT with SIB to tumor bed. In our analysis, there was no existing consistency between the displacement of surgical clip and bony structure. Even the position of bony structure was corrected by image-guided procedure; the displacement of surgical clips was still not negligible (shown in Table 2).

In the effort to find which group of patients was prone to more uncertainty of boost volume, we assessed many factors including age, tumor location, body mass index, volume of the breast, volume of the tumor bed, and breast thickness. Although younger woman tends to have dense breast tissue, which might render less target uncertainty during the treatment course, our result did not show the evidence. Based on this study, location of tumor, either in one of four quadrants or sub-areola, breast thickness, and breast volume affected the tumor bed displacement in AP direction for the univariate analysis, but none remained a predictor in the multivariate analysis. In this study, only body mass index did have statistical significance in multivariate analysis. In contrast, a study from Korea comprising 147 patients undergoing weekly IGRT of TomoDirect claimed that breast size was significantly associated with extensive set up error in multivariate analysis [18]. Of note, the definition of breast volume was different from our study. They estimated the bust and underbust size of patients with a measuring tape, while we used the CTV on the contouring as a reference.

In this study, patients having a higher BMI were stratified as high-variation group, and need more generous PTV-H margin of 7 mm, 10 mm, 10 mm in AP, CC, LR directions. Applying this PTV-H margin around the tumor bed might be a “double-edged sword”. Since the long term survival rate for early stage breast cancer was approaching to more than 90% [19], late complications in long-term survivors should always be a major concern. Darby et al. reported that the incidence of major coronary artery events increased linearly with the mean dose to the heart by 7.4% per gray, irrespective of underlying cardiac risk factors [20]. In addition, a prospective cohort study of about 30000 women showed that radiotherapy increased mortality from heart disease and lung cancer 10–20 years afterwards [21].

With respect to dose to organs at risk, a study investigating the effect of image-guided radiotherapy on the dose distributions in breast boost treatments showed a modest increase of doses to lung and heart when PTV margin of tumor bed expanded from 5 mm to 8 mm [22]. In theory, positional uncertainty of the boost volume could be mitigated at the expenses of receiving higher radiation dose to heart and lung. However, our study showed the dosimetric parameters of heart were not statistically different between the original and new plans for high- and low-variation groups. On the other hand, despite the dosimetric parameters of lung were generally higher in the new plans, the increment was limited. Therefore, more generous margin should be considered for patients with a higher BMI, and it can be safely applied through proper design of beam arrangement with advanced treatment techniques.

The limitation of the current study was that the impact of respiratory motion was not investigated. When applying respiratory-gated or breath-holding techniques; theoretically, the displacement of surgical clips would be mitigated. Nevertheless, a recent study [23] consisting of 58 patients with the analysis of setup errors during deep inspiration breath-hold (DIBH) disclosed the inter-fraction systematic error (∑) and random error (σ) was 1.4 mm and 1.7 mm respectively, which was comparable to those observed in their previous work for patients irradiated in free breathing (∑ = 1.1 mm, σ = 1.5 mm) [24]. Therefore, they concluded that the main benefit of the DIBH is to separate the heart from the target rather than irradiating the target more accurately. Despite they investigated the position of the chest wall instead of the tumor bed, their result suggested that breathing control might impact little on setup error during radiotherapy. Additionally, the procedures of respiratory-gated or breath-holding techniques were time-consuming and not always suitable for all patients. In the era of precision radiotherapy, patients should be treated with technically acceptable plan with incorporating individualized strategy to mitigate treatment uncertainty. If cone-beam CT is not routinely available or the physician concerned about the substantial imaging dose, daily on-board images can provide an accurate, efficient, and cost-effective way when adequate margin was added to the tumor bed.

## Conclusions

In patients with breast cancer receiving adjuvant radiotherapy after conservative surgery, a higher BMI is associated with a greater positional uncertainty of the boost tumor volume. More generous margin applied to tumor bed should be considered, and the doses to heart and lung could be limited through proper design of beam arrangement with advanced treatment techniques. Notably, the CTV to PTV margins are dependent on reproducibility of the treatment setup, immobilization, and image guidance. The margin size must be based on the institutional protocol that governs the setup variability evaluated at the respective institution.
